# Richmond Hill

**Published:** 1875-03

**Authors:** 


### RICHMOND HILL.

Precisely seven miles from the East River, on the Southern Railroad of Long Island, is situated the beautiful village of Richmond Hill. A portion of the ground comprised in the town-site is high and rolling, while the depot stands on a broad, level plateau, which is elevated about sixty feet above high-water mark. The town is readily accessible by two steam railroads, from Williamsburg and Hunter's Point, and one line of horse-cars from Brooklyn. Broad carriage-drives also connect this new settlement with the densely populated regions along the river bank. It occupies forty-five or fifty minutes to reach this charming spot from Broadway by the Grand Street or Roosevelt Street ferries. Once on the ground, the visitor will feel amply repaid for the trouble of the brief journey. He will find himself environed by singularly attractive surroundings. If he ascends to the hill-tops—one hundred and forty feet above high-water mark—his eye will take in the waters of the Ocean on the one hand and those of the Sound on the other ; while around him is spread a panorama of rare beauty. The Hill proper, slopes southward, and is thus warmed and illumined at all seasons by the sun's rays.

Two hundred and eighty acres of land have been laid out in streets and carefully adorned, with the intention of securing for the village the best class of settlers. Several lots have been sold, and twenty or thirty fine houses erected. An Episcopal church edifice was begun in July, 1874, and was completed and consecrated early in December. It is a tasteful structure, capable of seating over two hundred persons, and is entirely free from debt. The Rector is the Rev. Mr. Kimber, who resides in the village. A fine two-story school-building furnishes accommodations for several hundred pupils, and is used as a public hall when occasion requires. The streets are broad, and are adorned with more than seven thousand forest trees. At several corners, wells have been sunk, which supply freely the choicest water. Handsome drive-ways wind around by easy grades to the hill-top, and intersect the various thoroughfares. The many adornments of nature have been intelligently supplemented by the skill of man.

Some fine residences have been erected, and every building has a picturesque air. The aim has been neatness and attractiveness rather than great cost. The architect of most of the houses is Mr. Van Dien, who resides here. His work has been most thoroughly done. The residences have been constructed for comfort and durability, while retaining the idea of symmetry and grace.

Our visit to this place was made on a pleasant winter day—the 22d of February of this year. The beautiful hillside and the lovely plateau at its foot were bathed in joyous sunlight, and we thought we never looked upon a lovelier spot. What it will be when the ice and snow have given place to carpets of green and overhanging foliage, it is difficult to conceive. Certain it is that a more attractive situation will be hard to find. As we visited the place to learn something of its healthfulness, for the benefit of our readers, we directed our inquiries to that end. We learned that the water used was from wells and cisterns. The wells are deep, and pass through soft loam and a thick stratum of coarse gravel. The water from this source is soft and pure. The under-ground cisterns, which are numerous and carefully built, receive the rain-fall, and are chiefly relied on by some residents. Drainage is perfect, and there are no standing pools, or low, wet spots, to hold stagnant water, and nothing apparent which could poison the water supply.

The air is pure, and responds promptly to the ozone tests. In fact, oxygen in its most active and vital form could scarcely fail to exist continually, as no factories are permitted to exhale those noxious gases which so surely deprive the air of its vitality. As the owners of this property have determined to set it apart for the homes of thoughtful, intelligent, cultured, and wealthy families, it is but natural that they should forever prohibit, by binding covenants, the existence of any obnoxious trade or business in the village, and that they should establish certain building regulations, designed to secure symmetry and elegance throughout this delightful suburb. The prohibition alluded to must secure the atmosphere from contamination, and perpetuate the existing high sanitary conditions. These must be looked upon as very important considerations by intelligent heads of families, who seek a healthful locality no less than one possessing refined social surroundings. That both exist here in high degree, and are certain to continue for all time, is, we think, apparent.

Malaria, inducing the intermittents —which are known as "chills and fever"—is wholly unknown here, as we are assured by all. Indeed, the proven presence of ozone in good quantity, is of itself sufficient to establish the healthfulness of the town in this regard. The two can not exist in common; for malarial exhalations exhaust the atmosphere of its ozone, leaving no trace of it for the inhabitants. The existence of ozone in any locality is the surest test of its freedom from malaria.

The tests for ozone have been carefully made, and often repeated. Mr. Casamajer, a well-known chemist, conducted the experiments in a most careful manner, and has certified to the facts. His letter, which gives the result of his experiments with the air of various localities, resulting in a comparison most favorable to Richmond Hill, will be published next month.

The opinion of an intelligent and accomplished lady, who is rearing a family of children here, will be of interest to mothers. Mrs. Lyman is well-known to the public, as the author of "Kate Hunnibee's Diary," and many papers on domestic economy. Her residence at Richmond Hill has extended through several years, and her testimony does not differ from that of other residents. To Mrs. Lyman's letter we cordially invite attention.

The property which is offered for sale at Richmond Hill, comprises several houses, ready for occupancy, and a number of lots, of various size and price. Details may be learned on applying at the office, No. 52 Broadway, New York.

